# Selective Room-Temperature Catalytic Decomposition of Hypochlorite to Oxygen and Chloride

**DOI:** 10.1021/acsomega.5c01711

**Published:** 2025-07-03

**Authors:** Edin Živalj, Blaž Belec, Matjaz Valant

**Affiliations:** Materials Research Laboratory, University of Nova Gorica, Vipavska 13, 5000 Nova Gorica, Slovenia

## Abstract

Thermochemical cycles for hydrogen production involve a series of reactions, with water decomposition as the net result. One of the reactions frequently encountered in these cycles is the reverse Deacon reaction, where chlorine reacts with water vapor to form hydrogen chloride at high temperatures. An alternative, lower-temperature approach involves introducing chlorine to liquid water, yielding hydrochloric acid and hypochlorous acid. The hypochlorous acid can be further decomposed to hydrochloric acid and oxygen by catalytically induced reaction. The catalytic activity of ruthenium and iridium oxide nanopowders, along with UV light, was investigated for the decomposition of hypochlorous acid. Using Raman spectroscopy and mass spectrometry, we demonstrated that both catalysts facilitate decomposition to oxygen, while UV light promotes decomposition to chlorate ions. Ruthenium­(IV) oxide exhibited superior catalytic activity compared to iridium­(IV) oxide in both alkaline and acidic media. Both catalysts showed excellent recyclability, maintaining consistent activity after five consecutive tests. Structural stability was confirmed via XRD, Raman spectroscopy, and TEM analysis.

## Graphical abstract

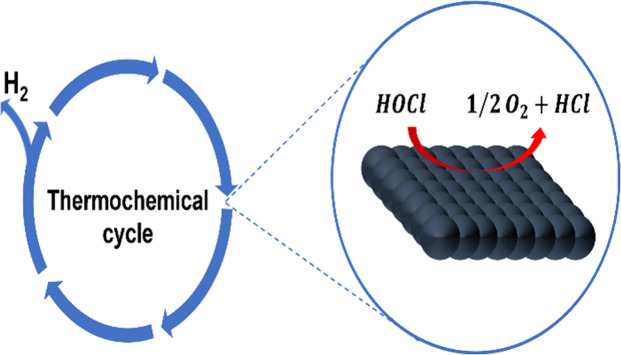

## Introduction

The combustion of fossil fuels releases greenhouse gases into the atmosphere, posing a serious threat to the environment and constituting one of the main causes of climate change. Coupled with the finite nature of fossil fuel reserves, the adoption of renewable technologies becomes inevitable. A sustainable alternative is hydrogen, which, nowadays, is mostly produced by reforming methane steam which uses natural gas and produces carbon dioxide. However, a more environmentally sustainable approach involves using water as the primary feedstock for hydrogen production. Various processes exist for producing hydrogen through water splitting, such as thermochemical cycles, electrolysis, photocatalysis, and processes involving microorganisms.

The thermochemical cycles for hydrogen production consist of a series of reactions where the net reaction is the decomposition of water. They provide a clean and sustainable path toward hydrogen with the potential for large-scale production and efficiency of more than 40%.^1,2^ More than a hundred cycles have been identified by General Atomics,^3^ yet only a handful have undergone further investigation during the last two decades.^4^ In pure thermochemical cycles, water and heat are the only two inputs. Hybrid cycles combine thermal with another form of energy, such as electricity. All other materials used in these cycles are recycled within. Most of the known cycles today involve at least one reaction that requires a temperature in the range of 500–1500 °C.^5^ To ensure these processes are considered carbon-neutral, the required heat must come from renewable energy sources such as nuclear,^6^ solar,^7^ or industrial waste heat.^8^ Finding a catalyst for the high-temperature reactions would reduce the required energy input, thus bringing these cycles one step closer to commercialization.

The reverse Deacon reaction 1 is the high-temperature reaction of cycles such as Cu–Cl,^9^ V–Cl,^10^ and Fe–Cl.^11,12^

Cl2(g)+H2O(g)↔2HCl(g)+1/2O2(g)T>600C° 1

Low conversion rates and high temperatures required for the reverse Deacon reaction significantly hinder large-scale implementation of these cycles.^5^ Another challenge to address is the recombination of hydrochloric acid and oxygen during the cooling of the products.^13^ Replacing a one-step reverse Deacon reaction with a two-step process that involves magnesium chloride can reduce the reaction temperature to 450–550 °C, which is still relatively high.^14^ Another yet unexplored way, is to convert Cl_2_ to HCl through reactions in liquid water.

Dissolving chlorine in water will give rise to a few different species according to the equation below:^15^

$$\begin{array}{ll} {\mathrm{Cl}}_{2(\mathrm{aq})}+H_{2}O_{\left(l\right)}↔H_{\left(\mathrm{aq}\right)}^{+}+{\mathrm{Cl}}_{\left(\mathrm{aq}\right)}^{-}+{\mathrm{HClO}}_{\left(\mathrm{aq}\right)} & K=3.9\times 10^{-4} \end{array}$$ 2

A small equilibrium constant (*K*) indicates that this reaction is shifted to the left side. From eq 2 it can be seen that chlorine (0) disproportionates in water to form hypochlorous acid (+1) and chloride ion (−1). Shifting the equilibrium toward products in eq 2 can be achieved by decomposing hypochlorous acid. Both hypochlorous acid (HClO) and hypochlorite ion (OCl^–^) can decompose through two different reaction pathways. The main decomposition product of the uncatalyzed reaction is chlorate ion with chlorine in +5 valence state:

$$3{\mathrm{HClO}}_{\left(\mathrm{aq}\right)}↔3H_{\left(\mathrm{aq}\right)}^{+}+2{\mathrm{Cl}}_{\left(\mathrm{aq}\right)}^{-}+{\mathrm{ClO}}_{3(\mathrm{aq})}^{-}$$ 3

$$3{\mathrm{OCl}}_{\left(\mathrm{aq}\right)}^{-}↔2{\mathrm{Cl}}_{\left(\mathrm{aq}\right)}^{-}+{\mathrm{ClO}}_{3(\mathrm{aq})}^{-}$$ 4

They can also decompose directly to oxygen, which is known as the minor side reaction:

$$2{\mathrm{OCl}}_{\left(\mathrm{aq}\right)}^{-}↔2{\mathrm{Cl}}_{\left(\mathrm{aq}\right)}^{-}+O_{2(g)}$$ 5

$$2{\mathrm{HClO}}_{\left(\mathrm{aq}\right)}↔2H_{\left(\mathrm{aq}\right)}^{+}+2{\mathrm{Cl}}_{\left(\mathrm{aq}\right)}^{-}+O_{2(g)}$$ 6

Reactions 3 and 4 are more favorable during uncatalyzed decomposition^16–18^ and are catalyzed by Cr­(VI) species^19,20^ and Te­(OH)_6_.^21^ The decomposition according to the reactions 5 and 6 occurs in the presence of oxides of Ni and Co.^22^ In this paper, the exact form of these metal oxides is not specified as the authors simply added chloride salts of these metals into an alkaline hypochlorite solution, which led to the precipitation of oxides or hydroxides. In addition, the effect of the additional chloride ions has not been considered as they are known to catalyze hypochlorite decomposition.^23^ The same paper also mentions Cu but no precipitate was formed after mixing copper chloride with an alkaline solution, yet, hypochlorite still got decomposed to oxygen and chloride ions.^22^ In this case, ionic Cu hydroxide species are responsible for the catalytic decomposition of hypochlorite.^24–26^ Kim et al.^27^ synthesized metal oxides, hydroxides, and oxide-hydroxides of Ni, Co, Cu, and Fe, and compared their activities in hypochlorite ion decomposition. Among the materials studied, Ni oxide-hydroxide exhibited the highest activity while Fe species practically did not exhibit any activity toward hypochlorite decomposition. Seminko et al.^28^ investigated the mechanism of hypochlorite decomposition by ceria, but it exhibited low activity, requiring 120 h to decompose 1 mL of a 2 mM hypochlorite solution at a ceria concentration of 0.1 g/L.

For the formation of a solution of hydrochloric acid with very low pH through the reverse Deacon reaction (eqs 1, 5 and 6), the catalyst must be stable in such an acidic medium. However, the oxides of Ni, Co, Fe and Cu readily dissolve under these conditions and cannot be employed in this process. Iridium compounds have shown activity toward hypochlorite decomposition, but the actual structure of a catalyst remains unknown as the authors simply mixed hexachloroiridate­(IV) ion with hypochlorite ion solution.^29^ Another paper describes a similar experiment with IrCl_3_ but, again, without characterizing the formed precipitate.^30^ In both papers, it was reported that the resulting iridium compound catalyzes the decomposition to both chlorate and oxygen. Macounová et al. investigated the mechanism of hypochlorite oxidation on RuO_2_-based electrodes and concluded that, regardless of the electrode material, the dominating product of oxidation is oxygen.^31^ Both RuO_2_ and IrO_2_ are not readily dissolved in hydrochloric acid, which makes them suitable candidates for catalyzing HOCl decomposition under low pH conditions. Ultraviolet light can also be used to decompose both hypochlorite and hypochlorous acid.^32^ Free radicals generated during UV light exposure complicate product selectivity. In aqueous chlorine solutions, this can lead to the formation of byproducts such as chlorate and perchlorate.^33^

The purpose of this study is to address the limitations of current chlorine-based thermochemical cycles for hydrogen production by exploring an alternative method to convert chlorine gas to hydrochloric acid through a direct reaction with liquid water at ambient conditions. This research investigates the hypochlorite decomposition pathway that results in the formation of chloride ions and the release of O_2_. We aim to identify and characterize catalysts that are both effective in acidic environments and capable of promoting selective decomposition. This approach aims to enhance the efficiency, sustainability, and commercial viability of hydrogen production using thermochemical cycles.

## Experimental Part

### Materials

Sodium hexachloroiridate­(III) hydrate (Na_3_IrCl_6_·*x*H_2_O) was purchased from Sigma. Ruthenium­(III) chloride hydrate (RuCl_3_·*x*H_2_O), 99.9%, and Sodium nitrate (NaNO_3_), 98+% were obtained from Alfa Aesar. Hydrochloric acid 32% was supplied from CARLO ERBA and Sodium hypochlorite solution, 12% Cl, stabilized, technical was purchased from Carl Roth. Doubly deionized water was used for the preparation of all solutions.

### Synthesis of IrO_2_ and RuO_2_

Both oxides were synthesized using the modified Adam’s fusion method.^34,35^ Initially, 0.25 g of RuCl_3_·*x*H_2_O or Na_3_IrCl_6_·*x*H_2_O, along with 5 g of NaNO_3_, were dissolved in 20 mL of deionized water. The solution was sonicated for 30 min and then mixed at room temperature for an additional 1 h. Subsequently, the solution was dried at 100 °C. The dried mixture was then transferred into a ceramic crucible and calcined at 450 °C for 1 h in an air atmosphere. After cooling to room temperature, the resulting oxide was filtered through a Büchner funnel with quantitative filter paper blue ribbon. Residual NaNO_3_ was removed by washing with deionized water and 0.1 M HCl solution. The dried black powders obtained from this process were used as the catalysts.

### Decomposition Experiments

First decomposition experiments were conducted on hypochlorite ion (OCl^–^) as it is the more stable form and commercially available. For the initial decomposition experiments, 0.05 g of catalyst was mixed with a 50 mL solution containing 3.5 mM OCl^–^_(aq)_. The catalyst was suspended by sonication of the reaction mixture for 20 s. The experiments not involving UV light were conducted in the dark. UV light-induced decomposition was carried out in a quartz reactor that was inside a UV chamber equipped with six ultraviolet lamps: three Philips Cleo 15 W lamps (emitting UVA light at ∼365 nm) and three S.N.E. RPR-2537A lamps (emitting UVC light at 253.7 nm). The lamps were arranged symmetrically within the reactor chamber to ensure uniform UV irradiation. Periodically, samples were taken, and the catalyst was separated by centrifugation at 13,400 rpm for 3 min. The spectrum of the supernatant was then recorded in a quartz cuvette. To investigate the effect of the temperature, these experiments were repeated using smaller amounts of catalyst (0.01 g) and the reaction mixture was put in an oil bath. Hypochlorite concentration was monitored using UV–vis spectroscopy due to OCl^–^_(aq)_ native absorbance peak around 295 nm.^36^ A calibration curve (Figure 1) was constructed using a stock solution of sodium hypochlorite, the concentration of which was determined by iodometric titration.^37^

**Figure 1:**
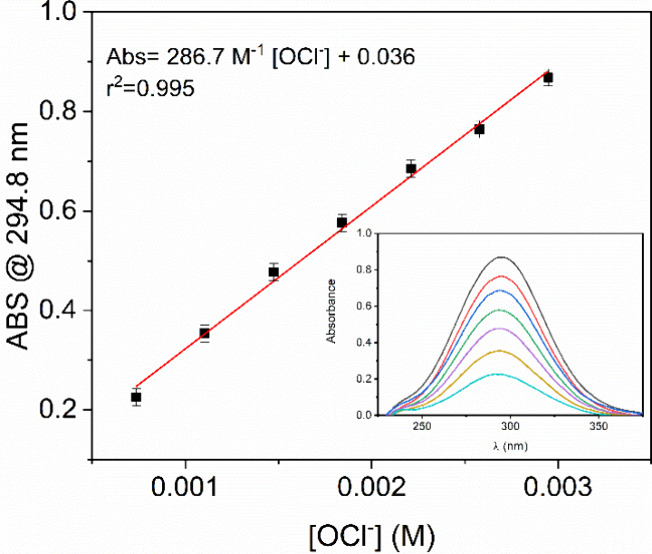
Dependence of the absorbance on hypochlorite ion concentration and the calibration curve equation. The inset shows hypochlorite ion UV–vis spectra for different concentrations.

It is known that the decomposition rate of hypochlorous acid is the highest at around pH 7.^23^ For that reason, the catalytic decomposition of hypochlorous acid (i.e., protonated hypochlorite ion) is too rapid to be monitored through sampling at specific time intervals. Instead, the reaction was conducted in situ, wherein 1 mL of HClO (concentration approximately 0.03 M) was placed in a quartz cuvette with a magnetic stirring. The HClO solution was prepared by lowering the pH of the hypochlorite ion solution to 5 with HCl_(aq)_. The absorbance at 267.5 nm was monitored continuously over time. A catalyst suspension of 0.2 g/L was prepared by dispersing the catalysts in pure water using an ultrasound bath. 200 μL of the suspension was added to the reaction mixture. This quantity was determined to be sufficient to observe the catalytic effect without significantly interfering with the UV–vis spectrum of HClO due to catalyst absorbance. After the reaction, the catalyst was recovered by filtration through the Büchner funnel and used the next day to prepare the suspension and repeat the experiment. Before each addition of the catalyst, the suspension was treated in an ultrasound bath. This was repeated for five consecutive days.

Both chlorate and hypochlorite ions have Raman-active modes, making Raman spectroscopy a useful method for monitoring reaction species over time.^38^ At room temperature (20 °C), 0.05 g of a catalyst was mixed with 50 mL of 0.28 M NaOCl solution. To obtain the spectrum, 300 μL of the catalyst-free solution was placed on a watch glass, and the spectrum was collected.

Since oxygen is released during the decomposition, as described by eqs 5 and 6, mass spectrometry (Pfeiffer Vacuum OmniStar GSD 350 O3) was used to analyze the gas evolving during the catalytic decomposition of hypochlorite ions. A suspension containing 0.05 g of catalyst in 25 mL of deionized water in a wide-mouth bottle with three inserts for screw caps equipped with threaded joints. The mixture was constantly purged with N_2_ and mixed with a magnetic stirrer. MS capillary was inserted into the bottle and after some time, 25 mL of a 0.06 M NaOCl solution was added to the reaction mixture through the third insert to initiate the reaction. The ion current for the 32 amu (oxygen) was tracked over time.

### Characterization

X-ray diffraction (XRD) patterns of synthesized oxides were collected using an X-ray powder diffractometer Rigaku MiniFlex600 with a D/tex Ultra detector equipped with a Cu Kα X-ray source (40 kV, 15 A). The morphology and size distribution of the particles were determined by field-emission transmission electron microscopy (TEM – JEOL JEM 2100UHR) operating at 200 kV and equipped with an Oxford X-Max80T energy dispersive X-ray spectroscopy detector (EDXS). For the TEM analysis, the particles were redispersed in water and deposited on a copper-grid-supported lacey carbon film. The size of the nanoparticles expressed as an equivalent diameter was determined from the TEM images, on which 200–300 particles per sample were accounted for the statistic using Gatan Digital Micrograph Software. The obtained data, representing the frequency count of the size distribution, were fitted using the single peak Gaussian fit mode.

Surface area measurements were carried out using an Anton Paar Autosorb iQ instrument equipped for both physisorption and chemisorption. For each analysis, 100–150-mg of sample was used. Prior to N_2_ adsorption at 77 K (liquid nitrogen bath), the samples were activated by heating at 120 °C for 24 h. Surface area was determined from the resulting isotherms using the multipoint Brunauer–Emmett–Teller (BET) model.

Raman spectra were collected using a Princeton Instruments IsoPlane SCT320 spectrometer. The system was coupled to an APE Research BA310MET optical microscope with a 50× objective lens for precise sample focusing and excitation. A laser with a 532 nm wavelength was used as the excitation source, with parameters optimized for two types of samples: oxide catalysts and liquid samples. For oxide catalysts, the laser power was set to 10 mW, with an exposure time of 20,000 ms per frame and 10 exposures per spectrum. For liquid samples, the laser power was increased to 50 mW, with an exposure time of 10,000 ms per frame and 10 exposures per spectrum. In both cases, the spectrometer was configured with a grating of 1800 g/mm (blaze wavelength: 500 nm) and a center wavelength of 900 cm^–1^.

UV–vis absorption spectra were recorded using a modular spectrophotometer setup. The system consisted of an Ocean Insight Ocean-HDX-XR miniature spectrometer connected to a cuvette holder via an optical fiber. The cuvette holder was illuminated by a DH-2000 dual light source (Ocean Insight) providing both deuterium and halogen lamps for coverage of the UV–vis range. A quartz cuvette with a path length of 1 cm was used for all measurements. Baseline correction was performed using water.

## Results and Discussion

### Catalyst Characterization

XRD patterns of the synthesized Ru and Ir oxides are presented in Figure 2. The diffraction lines of synthesized oxides correspond to a tetragonal RuO_2_ (PDF Card No.: 00-040-1290) (Figure 2, left), and tetragonal IrO_2_ (PDF Card No.: 00-015-0870) (Figure 2, right). The broadness of the peaks suggests that both oxides are nanopowders and the average crystallite size obtained using the Scherrer equation is 4 and 2 nm for RuO_2_ and IrO_2_, respectively. This is confirmed with TEM analysis where the particle size was determined to be ∼5 nm for RuO_2_ and ∼2 nm for IrO_2_. The particles of both oxides are roundly shaped (Figure 3). Figure 3c shows an HR-TEM image of a RuO_2_ nanoparticle, revealing a lattice fringe spacing of 0.32 nm, corresponding to the (110) planes of the tetragonal RuO_2_ structure (PDF Card No.: 00-040-129). The STEM-EDXS mapping (Figure 1a, inset) shows an increased Ru signal in the region containing agglomerated RuO_2_ nanoparticles. Similarly, Figure 3f presents an HR-TEM image of an IrO_2_ nanoparticle, exhibiting a 0.25 nm lattice fringe spacing that matches (101) planes of the tetragonal IrO_2_ structure (PDF Card No.: 00-015-0870). The corresponding STEM-EDXS mapping (Figure 1d, inset) shows an increased Ir signal over the IrO_2_ agglomerate.

**Figure 2:**
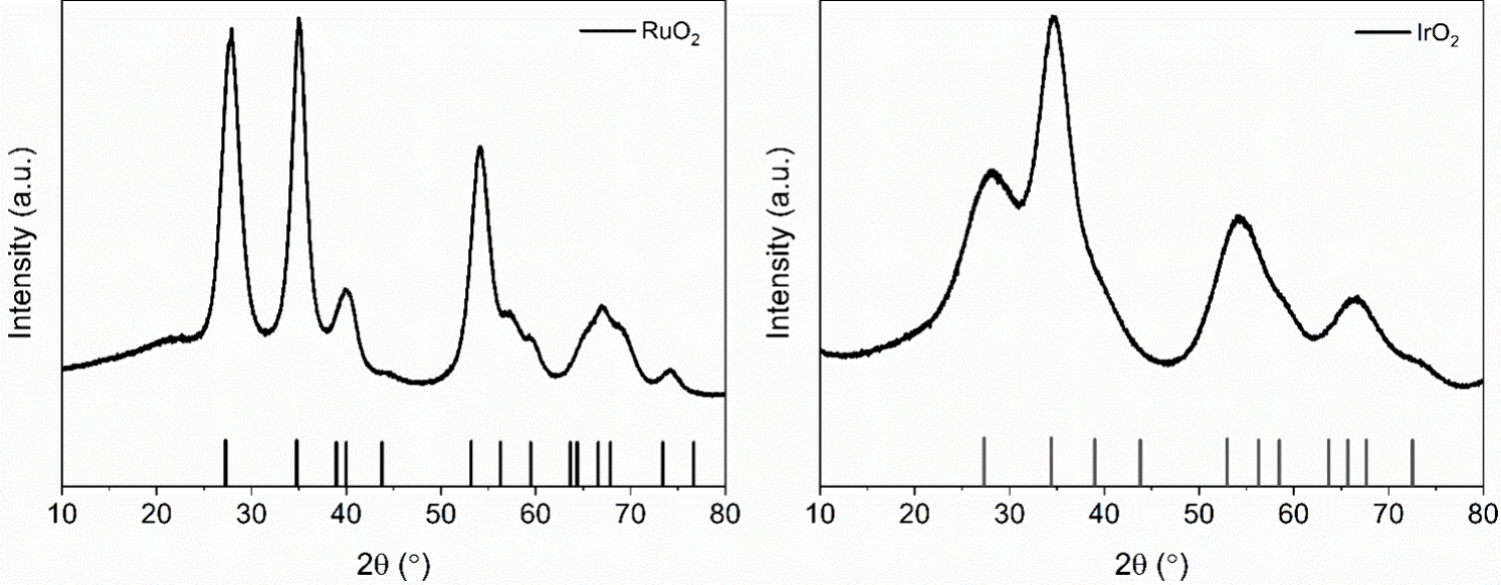
XRD patterns of synthesized RuO_2_ (left) and IrO_2_ (right). Characteristic peaks of RuO_2_ (PDF Card No.: 00-040-1290) and IrO_2_ (PDF Card No.: 00-015-0870) are presented as lines.

**Figure 3:**
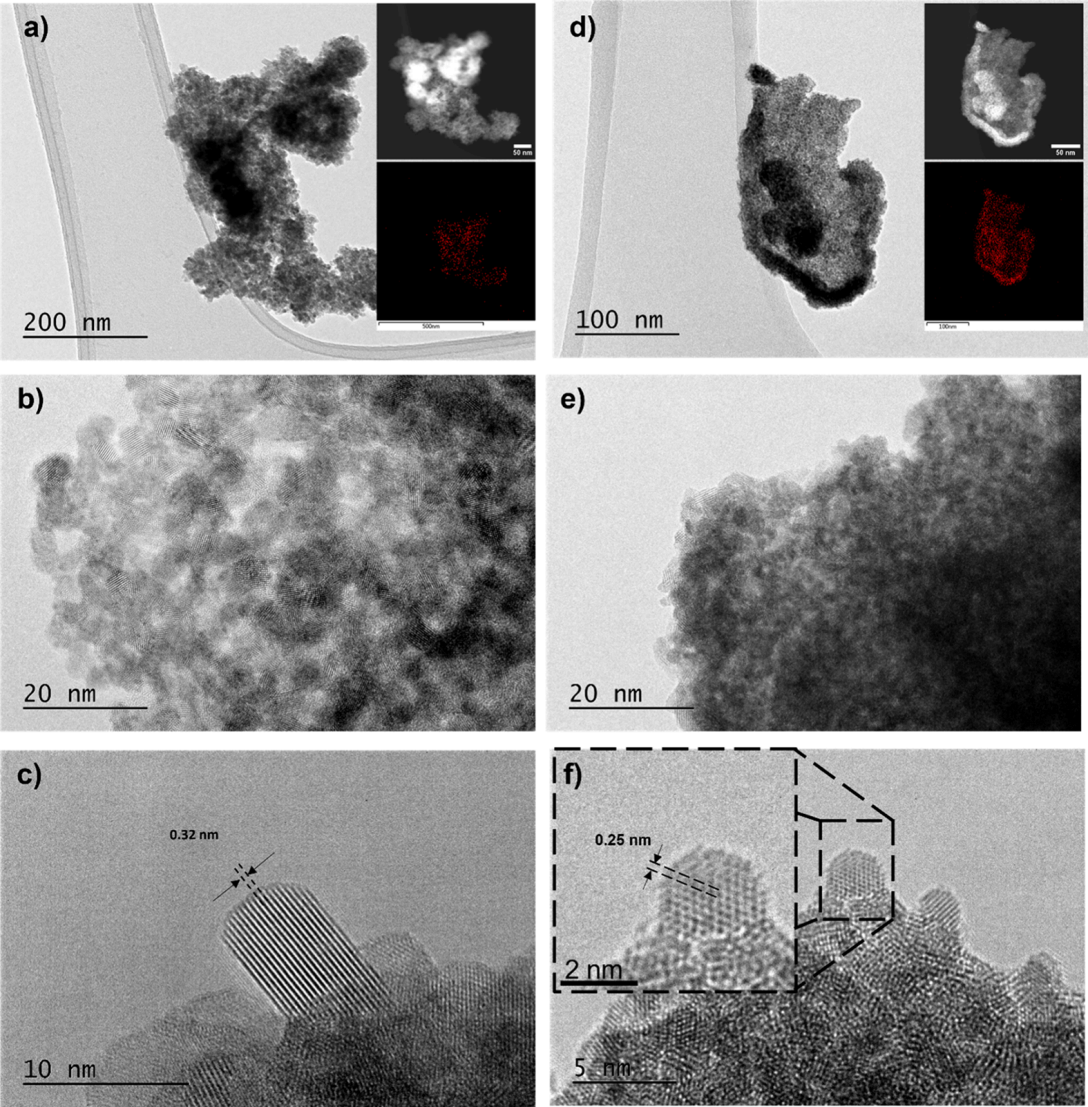
TEM images of synthesized RuO_2_ (a–c) and IrO_2_ (d–f). Images (a,d) include STEM-EDXS insets; (b,e) show higher magnification views; (c) shows an HR-TEM image of RuO_2_ with denoted lattice fringe spacing, while (f) shows an HR-TEM image of IrO_2_ with a close-up of a particle and corresponding lattice fringes.

Single-crystal RuO_2_ has three major Raman active modes, located at 528 cm^–1^ (E_g_), 646 cm^–1^ (A_1g_) and 716 cm^–1^ (B_2g_).^39^ All three modes are observed in the synthesized RuO_2_ (Figure 4, left) with peak positions at 494, 610, and 675 cm^–1^. A red shift in the peak positions of all three modes is evident in the synthesized oxide compared to the single-crystal RuO_2_. This shift, along with the broad peaks, suggests that the synthesized RuO_2_ is nanocrystalline which is in agreement with XRD and TEM analysis.^40,41^ Three Raman active modes of a single-crystal IrO_2_ are positioned at 561 cm^–1^ (E_g_), 752 cm^–1^ (A_1g_) and 728 cm^–1^ (B_2g_).^42^ Two bands, A_1g_ and B_2g_, overlap to form a single band in nanocrystalline structures of IrO_2_.^43–45^ The Raman spectrum of synthesized IrO_2_ has two bands, at 538 and 710 cm^–1^ which can be ascribed to E_g_ and combined A_1g_ + B_2g_ bands, respectively. The red shift observed in the synthesized IrO_2_ compared to the positions of the single-crystal peaks indicates that the material consists of nanoparticles, which, again, is in agreement with XRD and TEM analysis.^43^ The specific surface areas of the RuO_2_ and IrO_2_ nanopowders were determined through N_2_ adsorption–desorption (BET) measurements, yielding values of 162 and 315 m^2^g^–1^, respectively.

**Figure 4:**
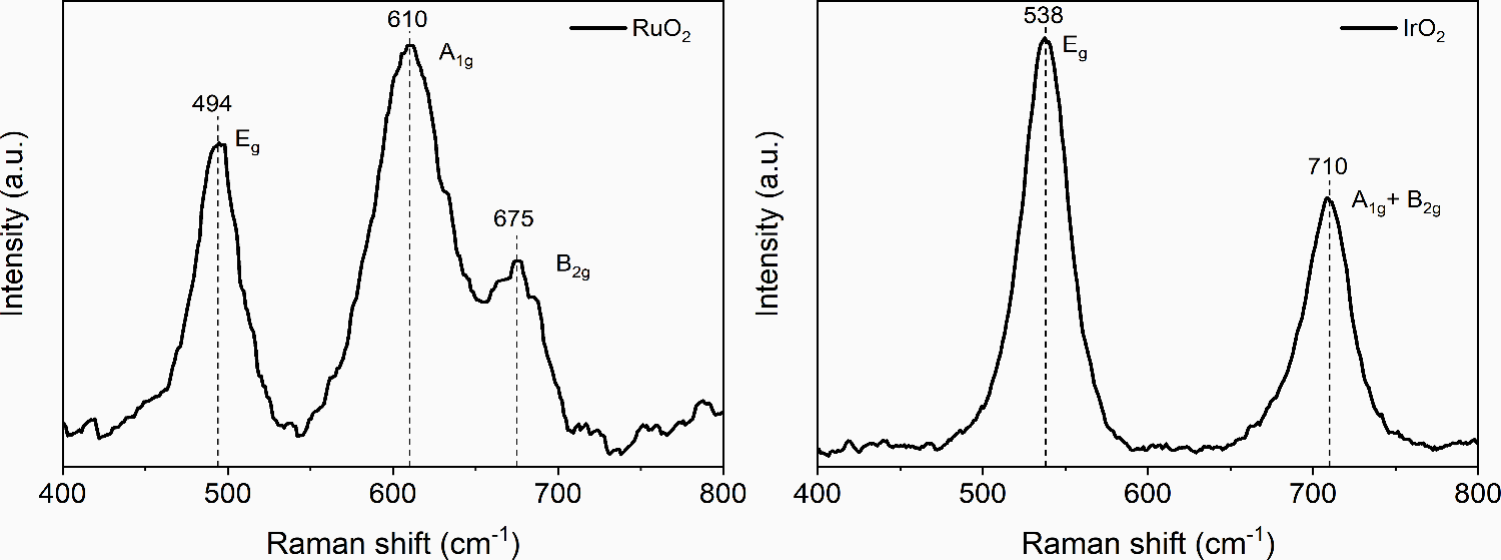
Raman spectra of synthesized oxides, RuO_2_ (left) and IrO_2_ (right) with their Raman-active modes and peak positions noted.

### Decomposition Experiments

Figure 5 shows that UV light and RuO_2_ have similar activity, while IrO_2_ exhibits poorer activity toward the decomposition of hypochlorite ions. It is also worth noting that during the experiments with RuO_2_ and IrO_2_, gas bubbles are observed to evolve from the reaction mixture. This is the first indicator that both oxides catalyze hypochlorite decomposition to oxygen. Although UV light and RuO_2_ demonstrate comparable activity in hypochlorite decomposition, the following analysis explains why UV-catalyzed decomposition is not appropriate for Cl_2_ to HCl conversion according to the reverse Deacon reaction

**Figure 5:**
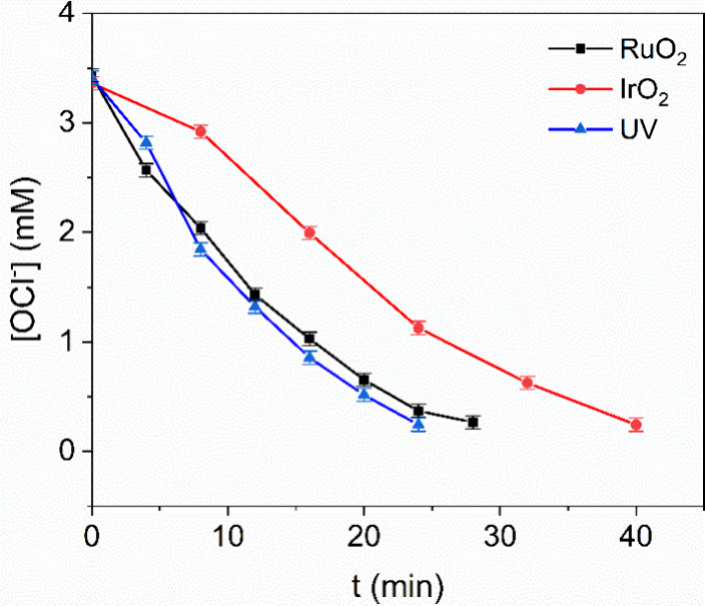
Hypochlorite ion concentration during decomposition reaction with different catalysts and UV light. At room temperature, 0.05 g of a catalyst was mixed with 50 mL of 3.5 mM NaOCl solution. The intensity of 294.8 nm absorption band was used to quantify the OCl^–^ concentration.

According to reactions 4 and 5, hypochlorite ions can decompose either to oxygen and chloride ions or disproportionate to chloride and chlorate ions. Since both chlorate and hypochlorite ions have Raman-active modes, Raman spectroscopy was used to monitor the concentration of these species over time. The hypochlorite ion is characterized by Cl–O stretching with a band at 700 cm^–1^. The chlorate ion has three bands: 468 cm^–1^ is assigned to antisymmetric bending, 602 cm^–1^ is assigned to symmetric bending, and 923 cm^–1^ to symmetric stretching of ClO_3_^–^.^38,46^ The initial spectrum (before the reaction started, *t* = 0 min) shows the chlorate ion band because the commercial solution of sodium hypochlorite always contains some chlorate ions due to its decomposition over time. During the reaction with RuO_2_ and IrO_2_ catalysts, an increase in the chlorate ion band is not observed, while the intensity of the hypochlorite band decreases over time (Figure 6a,b). This proves that no chlorate is formed during the reaction and indicates that oxygen and chloride are the reaction products. In contrast, the UV light-induced decomposition results in a noticeable increase in the chlorate ion band, (Figure 6c). Given that the primary objective of this research is to decompose hypochlorite ion/hypochlorous acid to oxygen and avoid disproportionation to chlorate, this observation eliminates UV light-induced decomposition as a viable option. For this reason, no further experiments with UV have been conducted.

**Figure 6:**
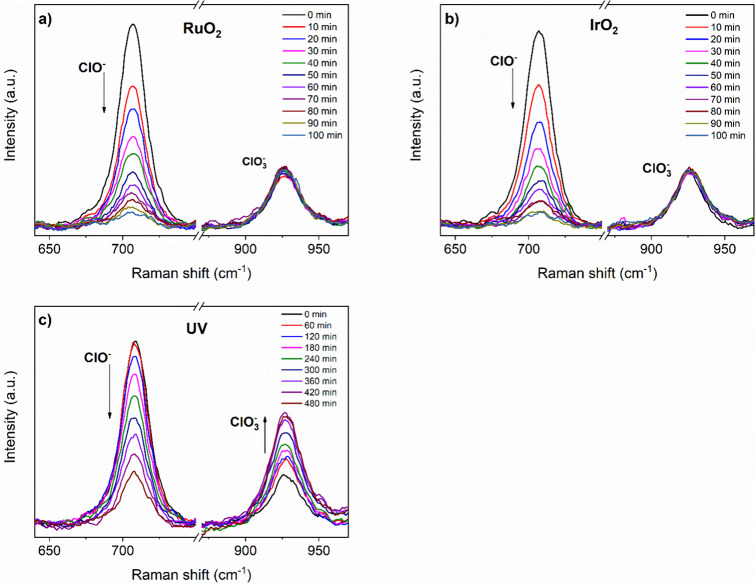
Raman spectra of the catalyst-free reaction mixture during (a) RuO_2_, (b) IrO_2_, and (c) UV catalyzed hypochlorite ion decomposition. At room temperature, 0.05 g of catalyst was added to 50 mL of 0.28 M NaOCl solution. Samples were periodically taken (time intervals noted on graphs), and the catalyst was separated by centrifugation before recording the spectrum.

The formation of oxygen during the catalytic decomposition of hypochlorite was confirmed using mass spectrometry. Upon adding sodium hypochlorite solution to a catalyst suspension, a noticeable increase in the ion current corresponding to oxygen (*m*/*z* = 32) was observed (Figure 7). The increase was more pronounced for RuO_2_ compared to IrO_2_, which is consistent with the decomposition experiments where RuO_2_ exhibited higher activity (Figure 4). On the contrary, the blank experiment with no catalysts showed no similar increase in ion current. Although Sandin et al.^30^ previously reported that RuCl_3_·*x*H_2_O and RuO_2_·*x*H_2_O, do not catalyze hypochlorite decomposition to chlorate nor oxygen, our results undoubtedly prove that RuO_2_ nanopowder effectively catalyzes hypochlorite decomposition to oxygen.

**Figure 7:**
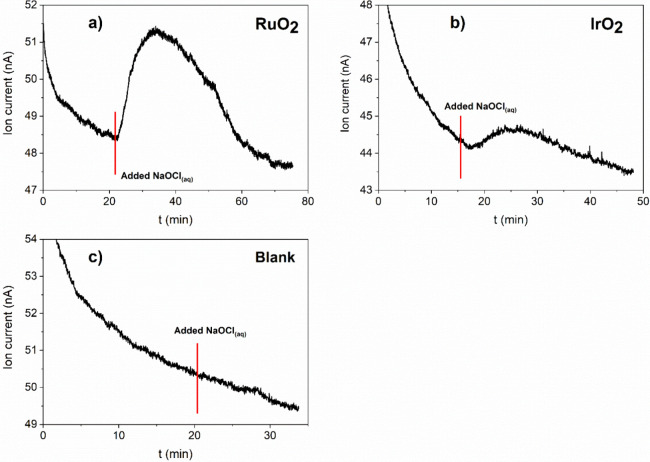
Change of ion current with time for the mass 32 amu (oxygen) during OCl^–^_(aq)_ decomposition with (a) RuO_2_, (b) IrO_2_, and (c) blank. A suspension containing 0.05 g of catalyst in 25 mL of deionized water was constantly purged with N_2_. At the indicated time (red line), 25 mL of 0.06 M NaOCl was added to the reaction mixture.

For both RuO_2_ and IrO_2_, a decrease in the amount of catalyst leads to a slower decomposition rate, consistent with expectations (Figure 8). Furthermore, the analysis reveals that RuO_2_ exhibits almost twice the activity of IrO_2_ across all catalyst concentrations.

**Figure 8:**
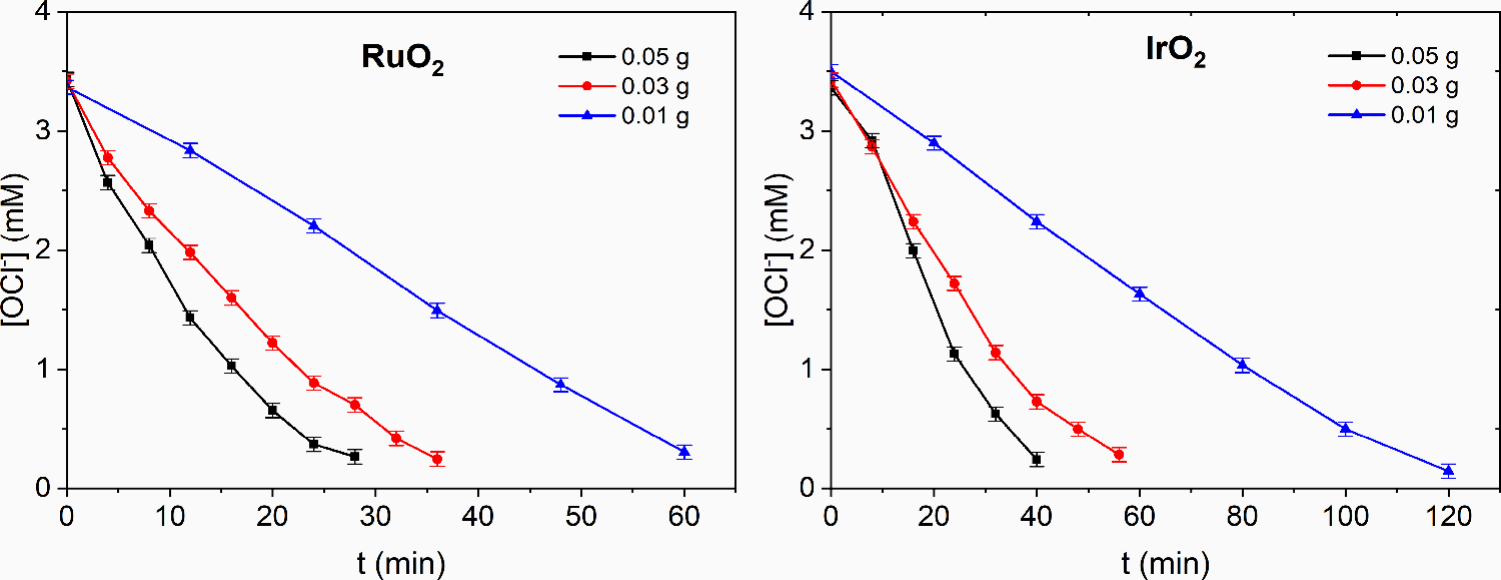
Effect of the catalyst load on the decomposition reaction kinetics. Three different amounts of both catalysts were mixed with 50 mL of 3.5 mM NaOCl solution at room temperature and samples were taken periodically for hypochlorite concentration analysis.

To investigate the influence of the reaction temperature on the catalytic decomposition of hypochlorite, the reaction temperature was varied from 20 to 50 °C. The initial concentration of hypochlorite ions was 3.5 mM and the catalyst 0.2 g/L. For both oxides, the decomposition rate increases with temperature as shown in Figure 9a,b. Elevating the temperature does not alter the decomposition pathway, as oxygen remains the product of the decomposition. However, for IrO_2_, a subtle increase in the chlorate band was observed (Figure 9d). This aligns with previous studies, which have shown that iridium compounds can catalyze both decomposition to oxygen and disproportionation to chlorate, although they favor oxygen decomposition.^47^ This was not observed at 20 °C because the disproportionation reaction is too slow and decomposition to oxygen prevails. For RuO_2_ the systematic increase in the chlorate band is not observed indicating the selective decomposition to oxygen (Figure 9c).

**Figure 9:**
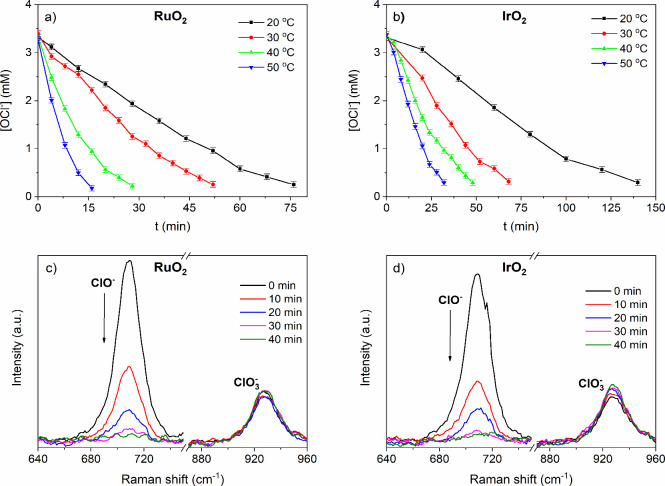
Effect of the reaction temperature on the catalytic hypochlorite ion decomposition for (a) RuO_2_ and (b) IrO_2_; a reaction mixture containing 0.01 g of a catalyst in 50 mL 3.5 mM NaOCl solution was held at four different temperatures, noted in the figures, in an oil bath. Raman spectra of the catalyst-free reaction mixture during (c) RuO_2_ and (d) IrO_2_ catalyzed hypochlorite ion decomposition; at 40 °C, 0.05 g of a catalyst was added to 50 mL of 0.28 M NaOCl solution. To obtain the spectrum, 300 μL of the catalyst-free solution was placed on a watch glass, and the spectrum was collected.

So far, experiments have been conducted with hypochlorite ion but we also studied the decomposition of hypochlorous acid (HClO). The starting pH was reduced to 5 to ensure complete protonation of OCl^–^ (p*K*_a_ = 7.53). The decomposition of HClO was monitored in situ with a UV–vis spectrophotometer. From the time scale in Figure 10, it can be seen that RuO_2_ is approximately three times more active catalyst than IrO_2_. This is in agreement with all the results from the decomposition of hypochlorite ions, which showed higher activity of RuO_2_. Despite IrO_2_ exhibiting approximately twice the specific surface area of RuO_2_, the higher catalytic activity of RuO_2_ indicates that this difference cannot be attributed to surface area effects, but rather arises from their intrinsic physicochemical properties. The spikes on the UV–vis absorbance plot are the result of oxygen evolution during the reaction. The bubbles evolving during the reaction cross the light path in the cuvette holder, causing absorption spikes. Accordingly, more spikes are present on the plot for RuO_2_, which is a more active catalyst, resulting in more intense oxygen evolution. The pH after the reaction was 2.8 which is attributed to the decomposition of HClO and release of H^+^ ions that were previously bound to HClO due to its weak acidic nature.

**Figure 10:**
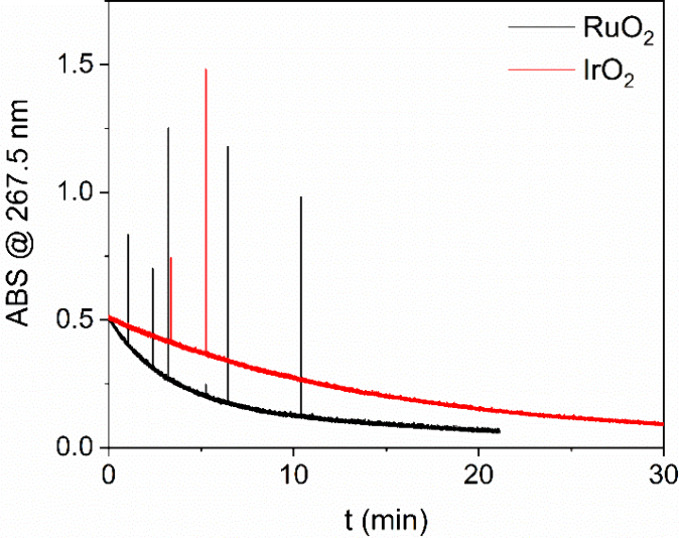
Absorbance over time of hypochlorous acid with RuO_2_ (black line) and IrO_2_ (red line) catalysts. A 1 mL 0.03 M HClO solution was placed in a quartz cuvette with a magnetic bar, and 200 μL of 0.2 g/L catalyst suspension was added.

To evaluate the recyclability of both oxides, the decomposition experiments were repeated five times using the same catalyst. After the first run, the catalyst was recovered and reused for subsequent runs. Before each repetition, catalyst suspension is treated with ultrasound to disperse the oxide particles. Both RuO_2_ and IrO_2_ maintained their activity throughout the process, as shown in Figure 11, where each run required a similar amount of time to achieve the same absorbance.

**Figure 11:**
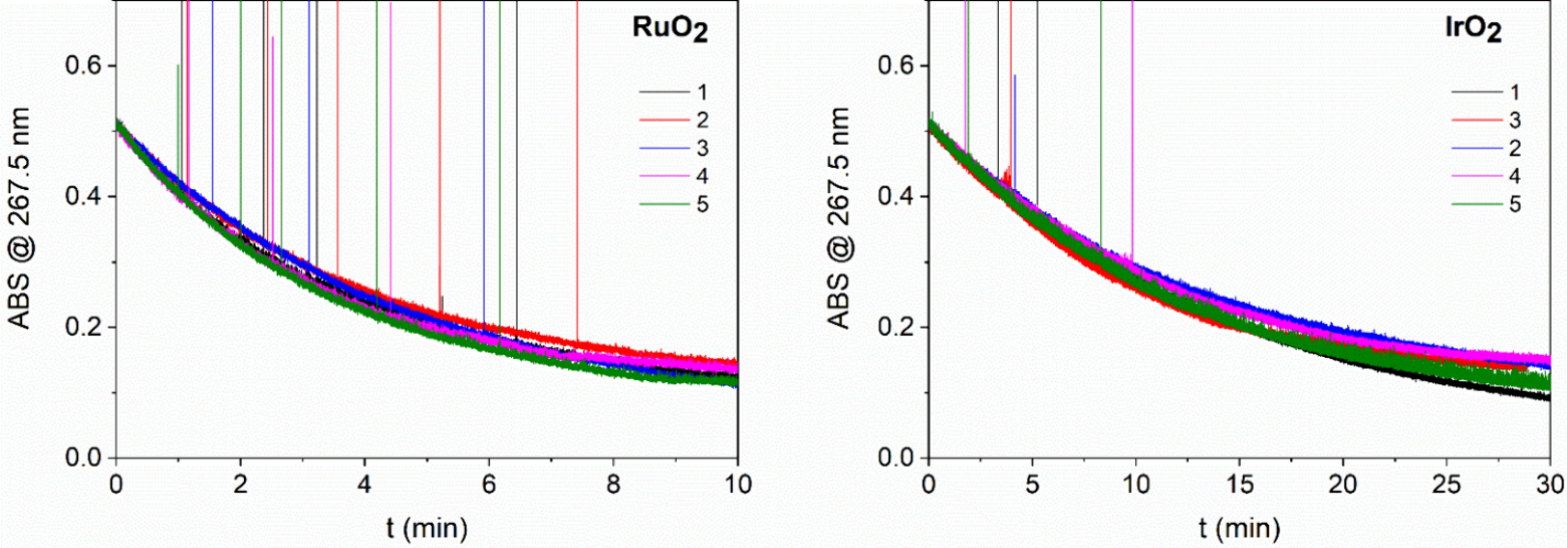
Absorbance at 267.5 nm of HClO/catalyst (RuO_2_ left, IrO2, right) mixture over time for five consecutive runs (run numbers are noted in the figures). At room temperature, 200 μL of 0.2 g/L catalyst suspension was added to 1 mL of 0.03 M HClO solution in the cuvette. After each run, the catalyst was recovered and used to prepare the suspension for the next run.

After the fifth run, both oxides were recovered and characterized to assess structural changes. XRD patterns of both oxides, recorded before and after the reaction, are overlaid in Figure 12a,b to compare peak positions, intensity, and shape. The overlapping patterns indicate no significant structural changes occurred during the reaction. Crystallite sizes of oxides were calculated using Scherrer equation and show no changes before and after the reaction. Size of RuO_2_ and IrO_2_ particles stayed ∼4 and ∼2 nm, respectively. Similarly, Raman spectra of both RuO_2_ and IrO_2_ before and after the reaction (Figure 12c,d) show no differences in peak positions or shape which confirms that both oxides remained in their initial structural forms.

**Figure 12:**
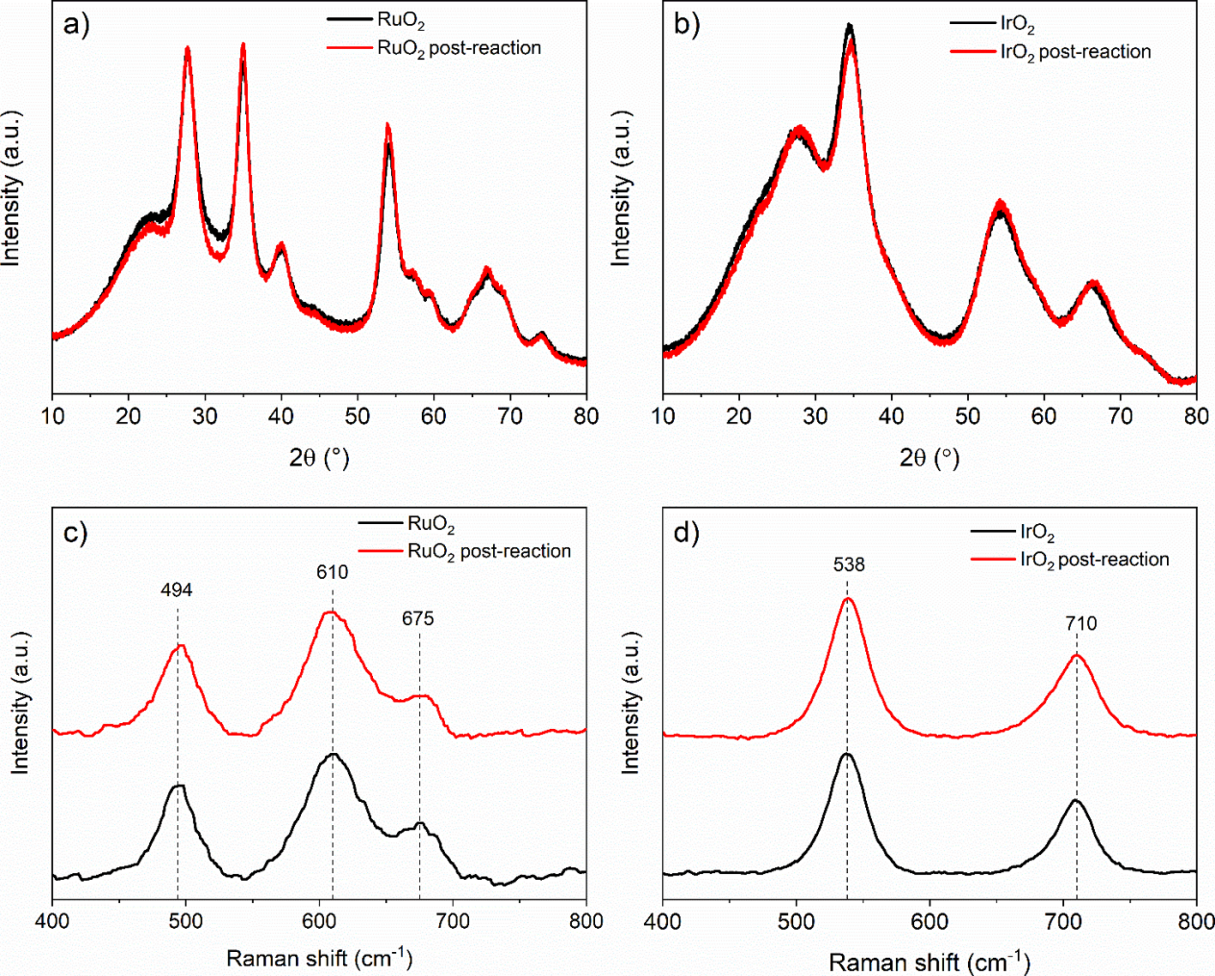
Overlaid XRD patterns of fresh (black) and recovered (red) (a) RuO_2_ and (b) IrO_2_ after the fifth run of the recyclability tests. Raman spectra of (c) RuO_2_ and (d) IrO_2_ before (black) and postreaction (red) are presented as stacked graphs.

In Figure 13, TEM images of RuO_2_ and IrO_2_ before and after the reactions are shown. There is no change in particle size of both oxides as they remained ∼5 and ∼2 nm for RuO_2_ and IrO_2_, respectively, which closely corresponds to the crystallite sizes obtained from XRD patterns. However, an amorphous layer around the particles has been observed in both samples after the recyclability tests (Figure 13b,e). Before each recyclability test, the catalyst suspension is sonicated. This ultrasound treatment likely causes the formation of amorphous layers. To confirm, the water suspensions of both oxides were subjected to 1 h of ultrasound treatment, and TEM images of the resulting samples were analyzed. These images also showed amorphous layers around the particles (Figure 13c,f). Thus, the amorphous layer is attributed to ultrasound treatment rather than the decomposition reaction itself. It is important to note that this amorphous layer did not hinder the catalyst’s activity toward hypochlorous acid decomposition as can be seen from Figure 11.

**Figure 13:**
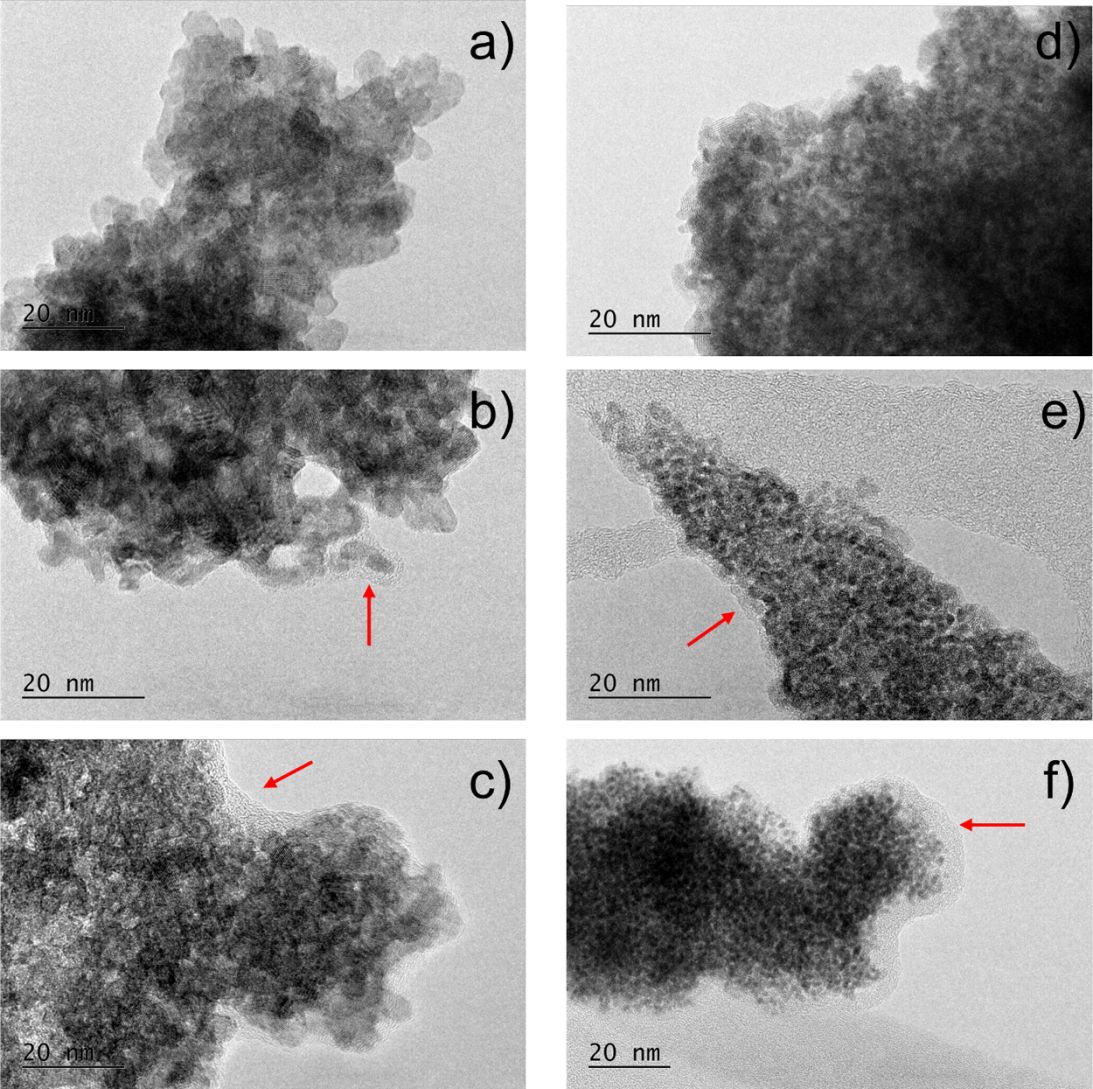
TEM images of RuO_2_/IrO_2_ (a/d), RuO_2_/IrO_2_ recovered after recyclability tests (b/e) and RuO_2_/IrO_2_ after ultrasound treatment (c/f). Red arrows are pointed toward amorphous layers formed around particles in images b, c, e, and f.

## Conclusions

Hydrogen storage and production continue to face challenges, due to technological or economical drawbacks of the current approaches. In the chlorine chemical cycles the bottleneck is reverse Deacon’s reaction that has to be applied for conversion of chlorine to hydrochloric acid. It requires high temperatures which reduces their energy efficiency An alternative involves dissolving chlorine in water and decomposing hypochlorous acid into hydrochloric acid and oxygen. This can be facilitated by catalysts or UV light. In this paper, we examined two catalysts that are chemically stable in highly acidic medium, i.e., IrO_2_ and RuO_2_. The Raman spectroscopy confirmed that chlorate ions do not form during the hypochlorite ion decomposition catalyzed by RuO_2_ or IrO_2_, unlike during the UV light-induced decomposition. The mass spectrometry revealed the evolution of oxygen during RuO_2_- and IrO_2_-catalyzed decomposition. An increase in the reaction temperature enhanced the reaction rate but did not alter the mechanism. RuO_2_ showed greater activity than IrO_2_. Both oxides retained their catalytic activity after five cycles, demonstrating very good recyclability. The XRD patterns, Raman spectra and TEM investigations confirmed that the reaction did not alter their structural integrity. These results show that at ambient conditions RuO_2_ has the potential to be used as a catalyst in thermochemical cycles where the conversion of chlorine to hydrochloric acid is required. It efficiently decomposes hypochlorous acid to hydrochloric acid and oxygen, resulting in a pure hydrochloric acid solution.

## Data Availability

All data supporting the findings of this study are included in the article.
